# Hybrid Bilayer PLA/Chitosan Nanofibrous Scaffolds Doped with ZnO, Fe_3_O_4_, and Au Nanoparticles with Bioactive Properties for Skin Tissue Engineering

**DOI:** 10.3390/polym12010159

**Published:** 2020-01-08

**Authors:** Julia Radwan-Pragłowska, Łukasz Janus, Marek Piątkowski, Dariusz Bogdał, Dalibor Matýsek

**Affiliations:** 1 Department of Physical Chemistry, Faculty of Chemical Engineering and Technology, Cracow University of Technology, 31-155 Cracow, Poland; jrpraglowska@chemia.pk.edu.pl (J.R.-P.); ljanus@chemia.pk.edu.pl (Ł.J.); pcbogdal@cyf-kr.edu.pl (D.B.); 2 Faculty of Mining and Geology, Technical University of Ostrava, 70800 Ostrava, Czech Republic; dalibor.matysek@vsb.cz

**Keywords:** electrospinning, hybrid polymer scaffolds, biomedical applications

## Abstract

Burns affect almost half a million of Americans annually. In the case of full-thickness skin injuries, treatment requires a transplant. The development of bioactive materials that promote damaged tissue regeneration constitutes a great alternative to autografts. For this reason, special attention is focused on three-dimensional scaffolds that are non-toxic to skin cells and can mimic the extracellular matrix, which is mainly composed of nanofibrous proteins. Electrospinning, which enables the preparation of nanofibers, is a powerful tool in the field of biomaterials. In this work, novel hybrid poly (lactic acid)/chitosan biomaterials functionalized with three types of nanoparticles (NPs) were successfully developed. ZnO, Fe_3_O_4_, and Au NPs were investigated over their morphology by TEM method. The top layer was obtained from PLA nanofibers, while the bottom layer was prepared from acylated chitosan. The layers were studied over their morphology by the SEM method and their chemical structure by FT-IR. To verify their potential in burn wound treatment, the scaffolds’ susceptibility to biodegradation as well as moisture permeability were calculated. Also, biomaterials conductivity was determined in terms of electrostimulation. Finally, cytotoxicity tests were carried out by XTT assay and morphology analysis using both fibroblasts cell line and primary cells. The hybrid nanofibrous scaffolds displayed a great potential in tissue engineering.

## 1. Introduction

Burn wounds are one of the most problematic skin tissue injuries. Burns that require medical treatment affect roughly 500,000 US inhabitants annually. Approximately 40,000 of them are hospitalized. The number of deaths causes from burn, which is almost 3500, proves that current treatment methods are insufficient [1]. Thus, novel methods of burn wound care must be developed. During thermal injuries treatment, doctors are focused on patients’ stabilization, infection prevention, and finally functional recovery, which starts with the inflammatory phase [1]. Skin is the largest human organ protecting the internal tissues from germs. Therefore, any damage to it may cause bacterial infection, blood loss, and disturbance of organs [1,2]. For many years, graft has been gold standard for full thickness skin burn recovery. However, there are many factors that may affect the healing process such as age, obesity, and chronic diseases, which may lead to graft rejection [1,2].

There are numerous biomaterials dedicated for skin tissue engineering based on synthetic and natural polymers. Nevertheless, commercially available scaffolds, which are mainly hydrogels, do not enable the three-dimensional structure of the extracellular matrix (ECM) restoration [2]. Thus, alternative methods of scaffolds preparation with predicted architecture have attracted scientists’ attention. One of the most promising pathways to obtain natural skin component equivalents is electrospinning. It enables the formation of polymeric nanofibers (NFs), which mimic the fibrous structure of collagen, laminin, and elastin [2,3]. Until now, different types of biopolymers were used to produce such biomaterials. Nevertheless, obtaining continuous and homogenous NFs is a problematic issue using pure protein solutions since numerous parameters affect the production process [2,4]. This can be overcome by preparing blends with other polymers, such as FDA-approved poly (lactic acid) (PLA) or poly (caprolactone) (PCL). However, the aforementioned materials, although they undergo biodegradation, when used in a too high concentration, they may lead to local inflammation of the surrounding tissues due to the pH decrease [5,6,7,8].

To improve potential nanofibrous scaffolds’ properties and their ability to stimulate tissue regeneration through their surface modification or by creating multi-layers scaffolds. It was shown that poly (vinyl alcohol)/poly (sodium alginate) hybrid material has very good cytocompability with skin cells and promotes their growth. However, these components do not undergo biodegradation under in vivo conditions and cannot be applied for full thickness skin regeneration [9,10,11,12,13]. This issue can be solved by adding ECM components that are immobilized due to covalent or hydrogen bonds formation or electrostatic interactions. For this purpose, various poly (saccharides) were used. Over the last few years, it has been also shown that one of the important parameters is nanomaterials characteristics is fibers alignment [4,5]. A right choice of the raw polymer, solvent, and potential enables the fabrication of a product with a designed and ordered morphology. For example, well-orientated fibers enable the preparation of nanomaterials with tensile modulus above 40 MPa which is comparable to healthy skin. Another important parameter is fiber diameter which may enhance cells proliferation. It has been proven that fibers with the diameters in the range between 250 and 300 nm promote human dermal fibroblasts better comparing to fibers of 1 μm. Spatial arrangements of nanosized fibers can induce integrin β1 signaling pathway leading to other cellular responses crucial in the healing process [4,5]. Nanofibers separate from a biomimical architecture provide nutrients delivery and very high surface area which contributes to cells adhesion and attachment. The morphology of nanofibrous materials gives also possibility of drugs incorporation which may promote healing process, prevent bacterial infections or increase vascular perfusion. Another way to upgrade NFs it to modify them with growth factors and enzymes [4,5,13].

A highly promising material for nanofibrous scaffolds production is chitosan, a chitin derivative that is known for its favorable properties, including biocompability and antioxidant and hemostatic activity [14,15]. Since it dissolves in acidic solutions, it can be used as a raw material for electrospinning in a native form or constitute a separate layer for a fibrous sheet. Due to presence of hydroxyl and amino groups, it may be modified by chemical or physical methods to create three-dimensional, porous structures. This polymer has a high chemical resemblance to one of the most common ECM components—proteoglycans and undergoes biodegradation under in vivo conditions [14,15,16,17].

Two-dimensional meshes prepared using electrospinning were found to restrict cellular penetration, which negatively affected the healing process. Currently used three-dimensional or hybrid nanofibrous materials enable cells infiltration due to the enlarged pores size and also promote their proliferation and extracellular matrix formation as well as endothelialization [4,5,6,13].

One of the most successful methods for nanofibers bioactivity improvement is their modification with various types of nanoparticles which affects their durability, antibacterial activity and may promote cells multiplication due to their conductive properties by DC current stimulation [17,18,19,20,21,22,23,24,25,26,27]. Nevertheless, such nanomaterials must be chosen carefully, since it has been shown that some NPs may undergo passivation process (Ag) or exhibit cytotoxicity (Cu) [20,24,25]. Another important issue associated with nanoparticles is the stabilizer being used, which may negatively affect biocompability [18,21,22]. Finally, NPs of too low size may penetrate cell membrane and lead to vacuoles formation followed by cell death [18,19]. Taking all together, the most promising types of nanoparticles are metal oxides such as ZnO [16,23], Fe_3_O_4_ [26], and metals like gold [23].

Zinc oxide is a metal oxide with semi-conductive properties that is used in biomedical applications due to its lack of cytotoxicity, antibacterial activity, and low price [23,24,25]. Moreover, Zno NPs have a large surface area and thus can act as efficient drug currieries. Some studies also show that their addition has a positive impact on sorption ability of the polymeric materials [24].

Iron (II, II) oxide nanoparticles have many favorable features like biocompability and electromagnetic properties. They are applied as contrast agents for magnetic resonance imagining (MRI), wound dressing components, or elements of drug delivery systems. What is important, this type of NPs is approved by the United States Food and Drug Administration (FDA), which makes them a great candidate for biomaterials preparation. It has been shown that Fe_3_O_4_ NPs enable polymeric scaffolds reinforcement, thus positively affecting the mechanical properties like the Young modulus [26].

Important feature of some NPs is their electrical conductivity or spectroscopic characteristics which enables cells stimulation using direct current or visible light. Application of external stimuli has been shown to significantly improve cells behaviors under in vitro conditions [23,24,25,26].

Silver nanoparticles are commonly applied in biomedicine, agriculture, waste-water treatment, catalysis and others, but it has been shown that their prolonged presence causes numerous side-effects including allergies or even cancer. They may also cause *argyria* and *argyrosis* and bioaccumulate [24]. Therefore, they should be replaced with more stable and less toxic metals such as gold, which is known of being chemically inert. Moreover, it has been reported that nanogold accelerates the wound healing process and promotes neovascularization and tissue granulation. They have also positive effect on cells proliferation. Finally, they have interesting optical and electrical properties [23].

Until now, it has been shown that scaffolds created from synthetic polymers exhibit moderate biocompability, whereas the choice of natural ones results in the preparation of materials with very poor mechanical properties and a too high degradation rate [6,7,8,9]. Thus, the most promising ones are composites. Numerous studies show that the use of various nanoparticles results in the significant improvement of scaffolds durability and biological activity [23]. Another method is the chemical modification of the polymeric structure [24]. It has been shown that the acylation of chitosan using fatty acid chlorides improves its mechanical properties and in vitro fibroblasts behavior [28].

In this article, a successful attempt was made to develop a new type of advanced nanofibrous, hybrid scaffold using the electrospinning method. For their preparation, an industrial-scale electrospinner was used to verify the possibility of commercial biomaterial applications. A series of scaffolds was prepared that were functionalized with three different types of nanoparticles known of their biocompability and positive effect on cells proliferation (ZnO, Fe_3_O_4_, Au). To enable and promote the neovascularization process, prepared nanofibers were embedded on the acylated chitosan porous layer. The NPs were prepared without the use of a stabilizing agent to improve their conductivity and minimize cytotoxicity. Ready materials were investigated for their chemical composition, morphology, electrical properties, and susceptibility to enzymatic degradation. Finally, their potential in burn wound treatment applications was determined through cytotoxicity study carried out on human primary cells (human dermal fibroblasts) and mouse fibroblasts cell line (L929). Also, phenotype studies were performed. The results showed that the proposed biomaterials have a great potential in the field of regenerative medicine and may significantly improve the healing process.

## 2. Materials and Methods

### 2.1. Materials

Chitosan of average molecular mass = 300,000 g/mol was purchased from PolAura (Dywity, Poland). Poly (lactic acid), acetone (analytical grade), acetonitrile, trifluoroacetic acid, NaCl, (CH_3_COO)_2_Zn 2H_2_O, NaHCO_3_, KCl, K_2_HPO_4_ 3H_2_O, MgCl_2_ 6H_2_O, KOH, HCl, CaCl_2_, chloroauric acid, Na_2_SO_4_, (CH_2_OH)_3_CNH_2_, methanol, ethanol, XTT assay, L929 mouse fibroblasts cell line, human dermal fibroblasts (HDF) primary cells, fibroblasts complete growth medium (FGM), Dulbecco’s Modified Eagle Medium (DMEM) with high glucose content, phosphate buffer solution (PBS), streptomycin/penicillin, trypsin, and FGF-2/basic FGF Protein were purchased from SigmaAldrich (Poznań, Poland). All other reagents were of analytical grade and purchased from SigmaAldrich.

### 2.2. Methods

#### 2.2.1. Nanoparticles Preparation

Briefly, to prepare ZnO nanoparticles, the NaOH solution was added to the zinc acetate solution at 60 °C until pH 11. The precipitate was filtrated off using Buchner funnel and washed out with distilled water 3 × 20 mL. Finally, the product was washed out with 5 mL of absolute alcohol (ethanol). Ready ZnO was followed by calcination at 300 °C for 60 min. Ferrimagnetic nanoparticles were obtained using FeCl_3_ 6H_2_O and Mohr salt ((NH_4_)_2_Fe(SO_4_)_2_. The salts were mixed, and the ammonia solution was instilled until it became black in color (pH = 10). The resulted particles were washed out with water until neutral pH. Gold nanoparticles were prepared using chloroauric acid as a metal source and citric acid as a reducing agent. To remove any by-products and salts, all NPs solutions were purified via dialysis process using MWCO 10,000–12,000 Da.

#### 2.2.2. Nanofibers Preparation

PLA nanofibers were obtained by preparing 10% solution of the polymer in acetone. The PLA purity grade was confirmed by FT-IR and XRF methods. For nanofibrous materials Industrial Electrospinning System RT Advanced was used purchased from Linari NanoTech (Pisa, Italy). Electrospinning was carried out at different potentials ranging from 20 to 40 kV. The distance between needle and collector was 10–20 cm, speed of polymer dosing 10–20 mL/h, 1000–1500 RPM. The spindle was covered with aluminum foil. Then, a nanofibrous mat was taken off from the foil and dried under room temperature until complete acetone evaporation.

#### 2.2.3. Chitosan Acylation

Chitosan (CS) acylation proceeded according to a previously described method using fatty acid chloride as an acylating agent [28]. Briefly, 0.5 g of the chitosan was placed in an acetone/TEA solution and irradiated for 30 min in a microwave reactor. Then, fatty acid chloride dissolved in 5 mL of acetone was instilled, and the reaction proceeded for 60 min. Ready products were washed out with methanol and hot water. Their purity was confirmed by FT-IR. Dried acylated samples were dissolved in 20 mL of aspartic acid solution (pH = 4), frozen at −20 °C, and lyophilized.

#### 2.2.4. Hybrid Scaffolds Preparation

To prepare bi-layer scaffold, nanofibers were immersed with methanol solutions of each NPs type and left for complete solvent evaporation. For further experiments, two types from the prepared mats were used that had a nanofibrous structure. Dried materials covered with nanoparticles were combined with the acylated chitosan by putting prepared 3D mats onto swollen hydrogel and lyophilized. The composition of each sample is given in Table 1.

#### 2.2.5. FT-IR Analysis

Fourier-transform infrared spectroscopy (FT-IR) analysis was conducted with FT-IR Nexus 470 Thermo Nicolet spectrometer purchased from Thermo Fisher Scientific (Waltham, MA, USA). For each measurement, an ATR diamond adapter was used. Before collecting the FT-IR spectra, each scaffold was freeze-dried.

#### 2.2.6. Morphology Study

Nanoparticles size and shape were investigated using a Transmission Electron Microscope, Jeol, (Peabody, MA, USA). To obtain photographs, each sample was dissolved in the methanol with analytical grade purity. Next, one drop of each solution was placed onto formvar-coated copper mesh, covered, and left to evaporate. The photographs were taken under HT = 80,000 V. The exposure time was 800 ms, whereas electron (e) dose was 2771.9 e/nm^2^. Pure nanofibers and those covered with the NPs were studied using an FEI Quanta 650 FEG Scanning Electron Microscope purchased from FEI (ThermoFisher Scientific, Hillsboro, OR, USA) using HV = 10 kV. The elemental composition by the XRF method was verified by an EDAX^®^ adapter.

#### 2.2.7. Conductivity Study

To measure the conductive properties of the prepared nanomaterials, an electrode (0.01 KCl) was immersed into the 10% PLA solution in acetone containing 1% of each NP type (ZnO, Fe_3_O_4_, Au) at 20 °C. For the study, an ELMETRON conductometer was applied from Zabrze, Poland. The cell constant was determined using a KCl 0.01 M standard solution.

#### 2.2.8. Water Vapor Permeability

Water vapor permeability (WVTR) was investigated by sticking freeze-dried samples to polystyrene well with area of 1 cm^2^. Each hole was filled with 5 mL of distilled water. The studies were carried out for 24 h at 37 °C. The WVTR value was measured calculating moisture loss by the following equation:

WVTR = (Wt − W0)/(tA)(g⋅m^−2^⋅d^−1^), (1)

where

W_0_—the initial weight,

Wt—the weight after time t,

T—the measuring time,

A—the area of the opening of polystyrene hole.

#### 2.2.9. Mechanical Study

The bilayer materials were cut into shapes measuring 50 mm × 10 mm and placed between cardboard grips. Then, the sample parts were conditioned in a saturated NaNO_3_ solution at 22 °C temperature and 45% relative humidity for 48 h. After conditioning, the samples were stretched with 100 N load cell (cross-head speed of 5 mm per min), and an initial grip separation of 20 mm. The average thickness of the chitosan hydrogels was determined by SEM microscopy. TS, Eb, and EM values were calculated with the obtained force deformation data using the following equations:

TS (MPa) = F/A,
(2)

where

TS—tensile strength,

F—peak force at failure (N),

Scaffold cross-sectional area (0.50 mm).

Eb = l_b_/l_0_ ∙1000, (3)

where

l_b_—elongation at break (mm),

l_0_—the original sample length (20 mm).

EM(MPa) = (∆F/A)/(∆l/l_0_). (4)

where

ΔF—the change in the force,

Δl—corresponding change in the sample length during initial linear deformation.

#### 2.2.10. Biodegradation

Biodegradation study was carried out by the enzymatic method using human lysozyme. For the study, each dried and weighed sample was placed in the 3D printed strainer, which was immersed in the simulated body fluid containing human lysozyme with the concentration typical for human body (mg/mL). After specified time intervals, the samples were taken out of the solution dried and weighed. The biodegradation progress was estimated by calculating weigh loss of each sample according to Equation (3):

BD = (W_0_ − W_t_)/W_0_ · 100%, (5)

where

BD—biodegradation degree,

W_0_—the initial weight,

W_t_—the weigh after time.

#### 2.2.11. Cytotoxicity and Electrical Stimulation of Cell Proliferation Study

Cytotoxicity study was carried out using a 2,3-Bis-(2-methoxy-4-nitro5-sulfophenyl)-2H-tetrazolium-5-carboxanilide salt assay, which provides data about the number of living cells through the colorimetric reduction of XTT, as well as the observation of cells morphology under inverted microscope under 40× and 100× magnification (Delta Optical IB-100, Planeta Oczu, Zielona Góra, Poland). For the study, L929 cell line and human dermal fibroblasts were used. The culture was conducted for 48 h (L929) and 168 h (HDF) under standard conditions (95% CO_2_ concentration, high humidity, 37 °C) using DMEM medium changed every 48 h. For XTT assay measurements, we used a UV-Vis spectrophotometer Agilent 8453 (Santa Clara, CA, USA). The electrical stimulation study was carried out according to procedure described by other researchers [29]. Briefly, the HDF and L929 cells were stimulated by a direct current of voltage of 50 mV for 3 h using platinum electrodes immersed inside of the cell culture flask.

#### 2.2.12. Phenotype Study

To determine the effect of the biomaterials on fibroblasts phenotype, a phenotype study was carried out. The presence of the basic fibroblast growth factor in the cell culture medium was determined using high pressure liquid chromatography (HPLC) according to the procedure described by Sluzky et al. [30]. A standard commercially available bFGF was used. The analysis was carried out using a Varian 9300 chromatograph, (Mulgrave—Victoria, Australia) equipped with autosampler. For the study, bFGF was denatured in the solution—mobile phase containing acetonitrile (50%) and trifluoroacetic acid (0.1%). The ratio of TFA/ACN was 1.7. Reverse phase (RP) Water symmetry 100 mm, 4.6 μm C18 column was used. For the analysis, all HPLC solvents were filtered using 0.22 μm membrane filters.

## 3. Results and Discussion

### 3.1. FT-IR Analysis

**Figure 1:**
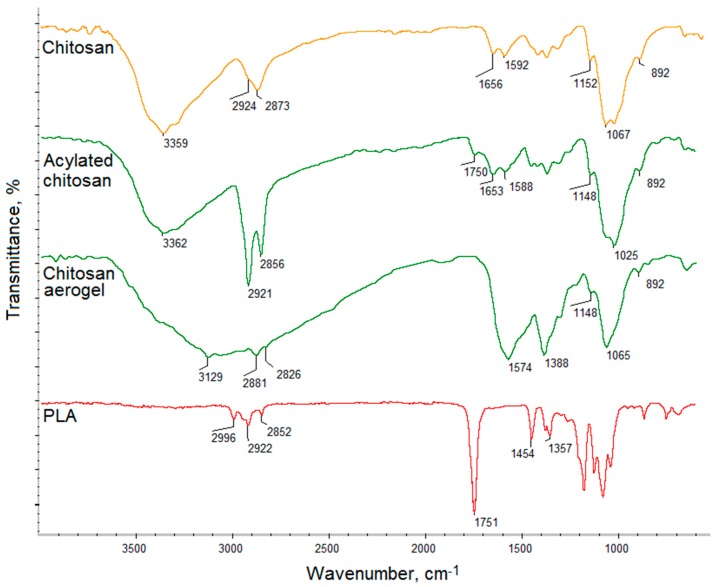
FT-IR spectra of the raw chitosan, acylated chitosan, chitosan aerogel, and PLA.

Figure 1 presents FT-IR analysis of raw chitosan and ready scaffolds components: chitosan aerogel prepared using *L*-aspartic acid and poly (lactic acid) nanofibers. As aforementioned, the CS layer was obtained from an acylated biopolymer, which was further turned into a porous aerogel. FT-IR spectra show chitosan before and after acylation process using fatty acid chloride. The raw polymer exhibit bands coming from free functional groups such as hydroxyl at 3359 cm^−1^ and amino at 1592 and 1152 cm^−1^. The band at 1656 cm^−1^ comes from *N*-acetylaminoglucose mers, while the band at 1067 cm^−1^ corresponds to β-glycosidic bonds between chitosan mers and the band at 892 cm^−1^ is typical for glucopyranose rings. Finally, bands at 2924 cm^−1^ and 2873 cm^−1^ corresponds to -CH- and -CH_2_- groups. The spectrum of the modified chitosan shows bands of significantly increased intensity at 2921 cm^−1^ and 2856 cm^−1^, which proves acyl chains incorporation [28]. A new band at 1750 cm^−1^ can be noticed, which means that the acylation reaction occurred due to the formation of ester bonds between free hydroxyl groups of chitosan and carboxyl groups. O-acylation resulted in the chitosan structure modification without losing free amino groups responsible for favorable features of the polymer. The spectrum of the chitosan aerogel shows the chemical structure of the acylated polymer converted into porous material using amino-acid solution, the presence of which can be confirmed by the presence of a wide band at 3129 cm^−1^ and the increased intensity of NH_2_ groups 1574 cm^−1^. Figure 1 shows the spectrum of the nanofibers prepared from PLA, which contains some typical bands such as 1715 cm^−1^ coming from ester bonds between mers. There are no abnormalities in the PLA spectrum, which suggests that it did not undergo degradation, does not content non evaporated solvent leftovers, nor is contaminated [31,32,33].

### 3.2. Morphology Analysis

**Figure 2:**
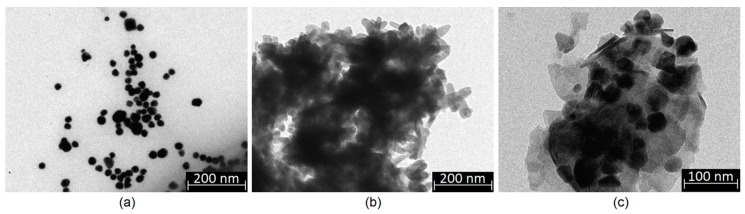
TEM images of the prepared nanoparticles: (**a**)—Au nanoparticles; (**b**)—Fe_3_O_4_ nanoparticles; (**c**)—ZnO nanoparticles.

Nanoparticles may constitute a powerful tool in nanomedicine and pharmacy due to their unique properties. However, their type must be chosen carefully due to parameters like their shape, size, presence of stabilizing agents, or surface charge [18,19,20,21,22]. Thus, preparation of biomaterials containing any type of NPs requires their complex analysis. Figure 2 shows TEM images of the prepared nanoparticles ((a)—Au; (b)—Fe_3_O_4_; (c)—ZnO). In the case of all nanoparticles, it can be seen that their size is above 10 nm, and thus, they will not penetrate the cell membrane and bioaccumulate due to the dialysis purification of the Au NPs citrate salts which are known to cause cytotoxicity. Obtained gold nanoparticles are of a round shape and are not covered with any matrix or salt crystals. Their size is approximately 20 nm. Fe_3_O_4_ nanoparticles prepared using two iron salts are of rectangular/oval shape without sharp edges which could damage cell membrane of dimensions 20 × 40 nm [18]. Their average dimensions are nm. ZnO nanoparticles are of 40 nm size and symmetric, polygon shape, which is typical for this metal oxide [24,25]. Again, no additional matrix may be observed, which proves their purity. The prepared NPs maintained their stability without using any stabilizers. It can be considered that all of the obtained functionalizing agents should exhibit typical physicochemical and biological properties.

**Figure 3:**
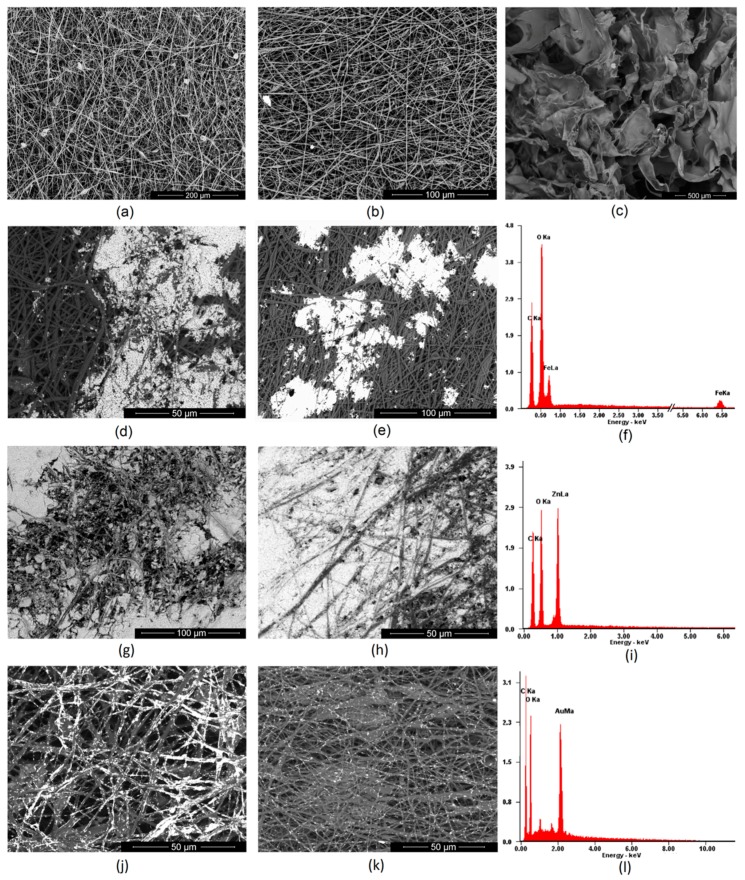
SEM images of the prepared nanofibers: (**a**)—pure PLA nanofibers obtained under 30 kV; (**b**)—pure PLA nanofibers obtained under 35 kV; (**c**)—acylated chitosan; (**d**)—PLA nanofibers obtained under 30 kV covered with Fe_3_O_4_ nanoparticles; (**e**)—PLA nanofibers obtained under 35 kV covered with Fe_3_O_4_ nanoparticles; (**f**)—XRF analysis of the surface composition (**g**)—PLA nanofibers obtained under 30 kV covered with ZnO nanoparticles; (**h**)—PLA nanofibers obtained under 35 kV covered with ZnO nanoparticles; (**i**)—XRF analysis of the surface composition (**j**)—PLA nanofibers obtained under 30 kV covered with Au nanoparticles (**k**)—PLA nanofibers obtained under 35 kV covered with Au nanoparticles; (**l**)—XRF analysis of the surface composition.

Nanofibers have a three-dimensional architecture that mimics an extracellular matrix. Thus, they provide excellent conditions for cell adhesion and proliferation [4]. However, to accelerate the skin regeneration process, especially in the case of full thickness burns, some additional stimuli are required that affect fibroblasts responses leading to certain enzymes, growth factors, and other signaling biomolecules secretion [4,5]. Numerous studies carried out on nanofibers with different dimensions showed that their diameter as well as their mutual position are important parameters affecting their biological properties. Obtainment homogenous and continuous fiber requires matching the right parameters such as polymer concentration, solution viscosity, type of solvent, applied potential, distance of needle from collector, or rotation speed. Figure 3a,b presents PLA nanofibers prepared using acetone due to its high volatility and easy removal trough evaporation. The study showed that the optimal parameters for NFs obtainment for industrial scale are two potentials: 30 (Figure 3a) and 35 kV (Figure 3b), respectively, distance between the needle and collector 10 cm, rotating speed 1000 RPM, and PLA dosing 10 mL per hour. One may observe that both types of nanofibers have a diameter below 1 μm and have co-axial morphology. However, NFs obtained under 30 kV have average size of 200–300 nm, whereas NFs prepared under 35 kV have a diameter in the range between 200–100 nm. No lumps or beads can be observed. Thus, both types of the fibers meet the requirements for tissue engineering [3,4,31,32,33,34].

Figure 3c shows an SEM microphotograph of the acylated chitosan layer. It is known that, for new tissue formation, another important property of the material is its porosity, which enables new blood vessel formation and microcirculation development [27,28]. It can be noticed that the chitosan part of the scaffold is highly porous, and pores edges have a petal-like shape. The average pore diameter is around 300 μm, which will enable cells migration and ECM growth in three dimensions.

**Scheme 1:**
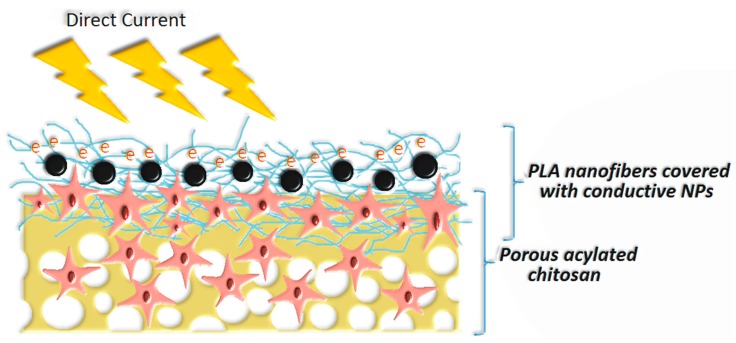
Proposed schematic structure of the prepared hybrid nanofibrous materials for skin tissue engineering.

To improve the bioactivity of NFs, various strategies may be applied, but poor wettability and low reactivity of PLA can make the modification process difficult [4,7,10,33]. In this article, three types of nanoparticles were used for poly (lactic acid) modification to increase their performance and give them conductive properties. Figure 3d–l shows PLA nanofibers covered with Fe_3_O_4_, ZnO and Au NPs and their elemental composition. It can be noticed that depending on the NPs type, the biomaterials morphology is different. Nanofibers covered with Fe_3_O_4_ NPs (Figure 3d,e) exhibit areas covered with nanoparticles which are partially aggregated. There are also smaller clusters visible. No NPs can be noticed on deeper situated fibers. The XRF Figure 3f of the nanomaterials confirms presence of the iron (II, III) oxide and lack of any metallic contaminants. Figure 3g,h shows nanofibers covered with another type of metal oxide nano-sized particles—ZnO. Due to a lack of intensive interaction in the contrary to ferrimagnetic NPs, the coverage is more uniform, and the degree of ZnO NPs deposition is significantly higher. It can be noticed that there are small differences in terms of coverage depth between sample CS-PLA-30-ZnO and CS-PLA-35-ZnO. In the case of samples prepared under 30 kV, ZnO nanoparticles can be spotted in the lower layers of the nanofiber due to the higher diameters of the resulted NFs as well as lower fibers density. In this case, it can also be observed that zinc oxide nanoparticles mostly adhere to each other and not the PLA surface. The XRF analysis in Figure 3i confirms the presence of ZnO and lack of metallic contaminants. The nanofibers coated with Au nanoparticles, which are visible at Figure 3j,k, exhibit a significantly lower degree of surface coverage. This can be explained by the fact that gold NPs can be also spotted at the deeper layers of the material, and their penetration depth is much higher comparing to metal oxide NPs. This could occur due to stronger interactions between metal and PLA. The TEM image of the Au NPs (Figure 2a) showed that these nanoparticles do not exhibit tendency to agglomeration. Therefore, they are best dispersed in the polymeric matrix. The degree of coverage is higher in the case of sample with higher fiber diameters. In this case, XRF analysis did not show any additional metal presence (Figure 3l). These results show that, depending on the type of NPs applied, prepared biomaterials can be used in superficial stimulation by a direct current as well as an in-depth current, depending on the needs of the final application. The proposed approach enables the preparation of scaffolds with tailored, conductive properties without applying toxic polymers such as poly (aniline) [35,36,37,38]. Schematic scaffold composition is shown at Scheme 1.

### 3.3. Electrical Properties of the Conductive Layer

**Figure 4:**
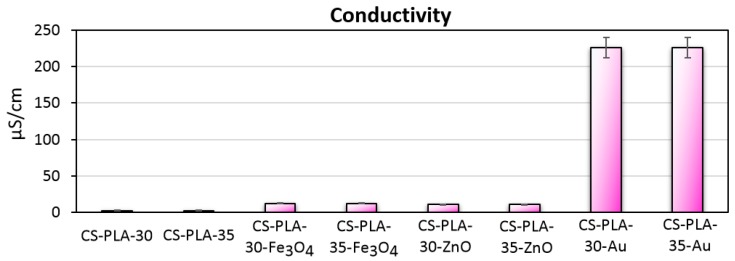
Conductivity of the prepared scaffolds.

There are numerous studies showing that the application of external stimuli improves various cell behaviors and accelerates tissue regeneration among which direct current stimulation can be named [28,33,34,35,36,37,38,39]. The use of DC has been proven to improve the proliferation of various cell types including L929. Thus, conductive biomaterials are now receiving a lot of attention. The most popular raw materials for such scaffolds’ preparation are poly (aniline), poly (pyrolle), and poly (vinyl pyrrolidone). However, their application may cause toxic effects under in vivo conditions. Therefore, alternative methods for biomaterials enabling direct current stimulation obtainment are of a high importance. Such strategies involve application of conductive nanomaterials. Figure 4 presents results of conductivity studies. Pure PLA has high resistance. Performed surface modification with the application of various NPs enabled increase of material conductivity. The highest κ = 226.0 μS/cm was obtained for gold NPs which is typical for metals (1% content). The values obtained for 1% ZnO (10.7 μS/cm) and Fe_3_O_4_ (12.4 µS/cm) are much lower due to the semi-conductive nature of metal oxides and band gaps of 3.3 eV and 2.84 eV, respectively. However, the biosafety for metal oxides is higher than for metals thus to increase the conductivity of the material, they can be used in the higher dose. Obtained results showed that the nanoparticles-doped scaffolds exhibit ability to be used for cells stimulation using direct current of low voltage [28,33,34,35,36,37,38,39].

### 3.4. Water Vapor Permeability of the Scaffolds

**Figure 5:**
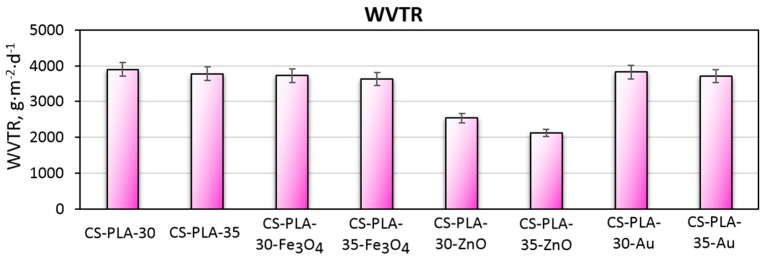
Water vapor transmission rate of the prepared scaffolds.

Water vapor permeability has been proven to be one of the crucial factors during wound healing process [40]. WVTR parameters differ in the case of healthy skin and damaged skin. The bad condition of this tissue requires higher moisture permeation for successful regeneration. Figure 5 presents the results of the WVTR tests. It may be observed, that prepared biomaterials have very good permeation for water molecules. Non-modified nanofibers WVTR is 3897 g·m^−2^·d^−1^ (30 kV) and (35 kV) g·m^−2^·d^−1^. The differences are cause by different fibers diameters along with fibers density. Samples modified with nanogold have almost the same permeability due to low coverage of the fibers and lack of self-aggregation between the NPs (3821 g·m^−2^·d^−1^ and 3712 g·m^−2^·d^−1^). The water vapor permeation for samples containing Fe_3_O_4_ NPs is 3722 g·m^−2^·d^−1^ and 3621 g·m^−2^·d^−1^. On the other hand, the WVTR parameter is significantly lower for samples containing ZnO nanoparticles (2542 g·m^−2^·d^−1^ and 2123 g·m^−2^·d^−1^ respectively) due to high coverage of the fibers structure as shown in Figure 3. ZnO has hygroscopic nature that can also affect water permeability. Thus, this type of scaffold would be more suitable for shallower wounds. The rest of the samples exhibit a water permeability rate that is typical for treatment of full-thickness skin [1,2,40].

### 3.5. Mechanical Properties Study

**Figure 6:**
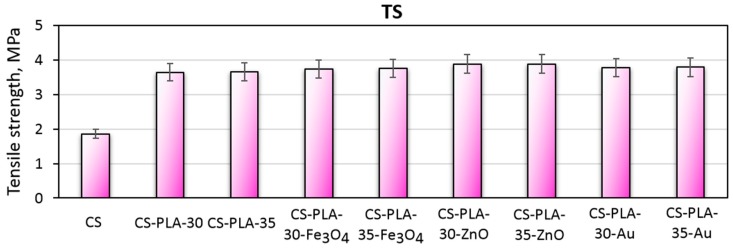
Tensile strength of the prepared scaffolds.

**Figure 7:**
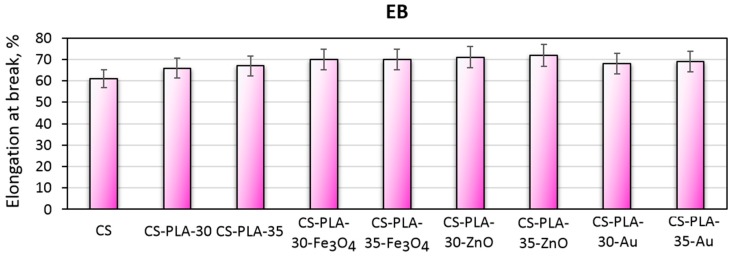
Elongation at break of the prepared scaffolds.

**Figure 8:**
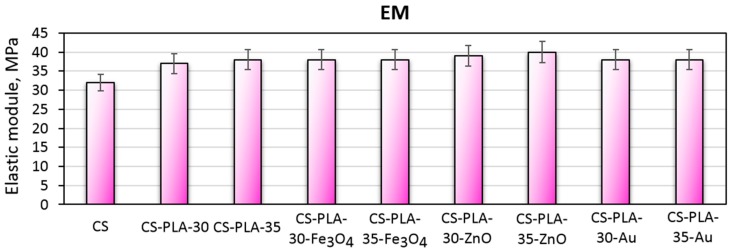
Elastic modulus of the prepared scaffolds.

Scaffolds dedicated to skin regeneration must provide mechanical support to newly formed tissue and maintain highly porous architecture to enable nutrients delivery and cells migration [27,28]. During the study, composite biomaterials were developed as a result of the combination of electrospinning, chitosan acylation and nanoparticles doping. The use of NPs, which are known to enhance mechanical durability, was expected to positively affect materials durability as well as acylated chitosan. The hypothesis was confirmed by the data given in Figure 6 showing tensile strength of the biomaterials corresponding to healthy skin [28,41]. The materials applicable in wound management should also be flexible. Figure 7 presents elongation at the break values for all samples. Again, it can be observed that the EB is dependent on nanoparticles type rather than PLA nanofibers density and diameter. It can be noticed, that nearly all samples have EB around 70%, which is typical for skin tissue. Finally, Figure 8 shows the elastic modulus, which is comparable for all samples. Such results can be assigned to the presence of elastic aliphatic chains incorporated inside linear chitosan as well as doping with nanoparticles [27,28,41,42].

### 3.6. Biodegradation Study

**Figure 9:**
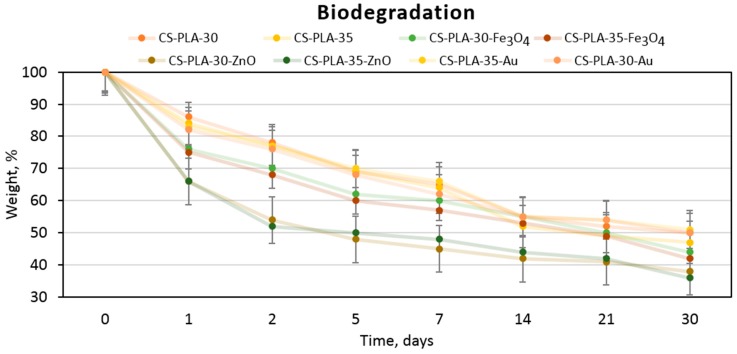
Biodegradability of the prepared scaffolds.

Scaffolds susceptibility of biodegradation under in vivo conditions is one of the crucial parameters for tissue engineering [4,5,10]. Figure 9 presents results of biodegradability tests carried out under in vivo simulating conditions. It may be noticed that all samples undergo a biodegradation process due to the weight loss observed in time (30 days). Due to the enzymatic hydrolysis, chitosan layer undergoes decomposition. The presence of this polymer which is known of its chelating properties acts as a buffering agent thus minimizing local pH decrease effect of PLA monomer (lactic acid) release into the medium. The highest biodegradation rate is noticed for first week. It can be noticed that the process did not achieve plateau phase and will be continued in time. Due to the high content of the acylated chitosan in the scaffold, this polymer degradation seems to determine the biodegradation speed and mechanism. It can be observed that the highest weight loss happens in the case of CS-PLA-30-ZnO and CS-PLA-35-ZnO samples (up to 64% loss), which can be explained by the fact that these scaffolds contained the highest amount of the nanoparticles that underwent the desorption process during the first days of biodegradation study. Such results correspond to other researchers’ data and confirms the applicability of the prepared fibrous materials in skin tissue engineering in terms of deep thermal burn injuries [27,28,29,30,31].

### 3.7. Cytotoxicity Study

**Figure 10:**
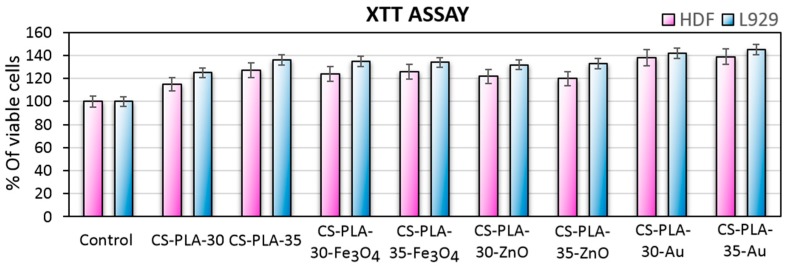
Metabolic activity of L929 mouse fibroblasts cell line and human dermal fibroblasts (primary cells).

**Figure 11:**
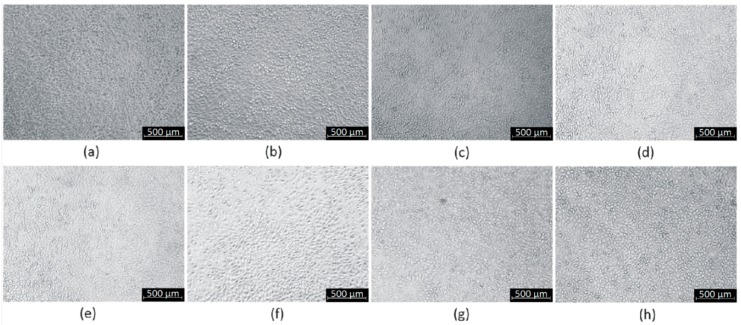
Cytotoxicity study carried out on L929 mouse fibroblasts (**a**)—CS-PLA-30; (**b**)—CS-PLA-35; (**c**)—CS-PLA-30-Fe_3_O_4_; (**d**)—CS-PLA-35-Fe_3_O_4_; (**e**)—CS-PLA-30-ZnO; (**f**)—CS-PLA-35-ZnO; (**g**)—CS-PLA-30-Au; (**h**)—CS-PLA-35-Au.

**Figure 12:**
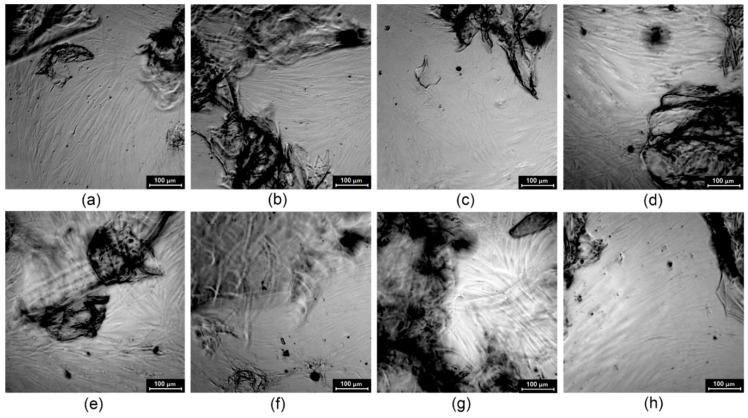
Cytotoxicity study on human dermal fibroblasts (**a**)—CS-PLA-30; (**b**)—CS-PLA-35; (**c**)—CS-PLA-30-Fe_3_O_4_; (**d**)—CS-PLA-35-Fe_3_O_4_; (**e**)—CS-PLA-30-ZnO; (**f**)—CS-PLA-35-ZnO; (**g**)—CS-PLA-30-Au; (**h**)—CS-PLA-35-Au.

To evaluate the potential in skin tissue engineering of the prepared nanofibrous scaffolds cytotoxicity study was performed by XTT assay (Figure 10) and morphology study method (Figure 11 and Figure 12). For this purpose, both mouse fibroblasts cell line (L929) and primary cells (human dermal fibroblasts) were used to determine dermis cells behavior in the presence of the scaffolds. Figure 10 presents the results of the cytotoxicity test, which is based on cell metabolic activity. It can be observed that all of the prepared scaffolds are non-toxic since the metabolic activity is higher than the control group, and they act positively on cells proliferation. Cell metabolic activity differs between samples. It can be noticed, that the best results are observed in the case of scaffolds containing nanogold (up to 145% for L929 and 139% for HDF). However, it should be noticed that the metallic nanoparticles non-toxic concentration is much lower compared to metal oxides, and the bioactivity effect can be noticed up to certain point [21,22]. The difference between samples regarding potential uses for fibers preparation (30 kV or 35 kV) is 9% for unmodified fibers, while for the samples containing nanoparticles, it is negligible. Such results show that the composition of the samples is suitable for dermis cells and do not affect negatively cell cycles or generate reactive oxygen species [21,22,23,24,25,26].

Figure 11 shows L929 cells cultured in the presence of scaffolds extract prepared according to ISO norm dedicated for biomaterials. It can be observed that, after 48 h of cell culture in the presence of prepared extract, uniform fibroblasts sheets are formed without abnormalities in their morphology. The results correspond to XTT assay obtained for human dermal fibroblasts and L929 and prove lack of cytotoxicity. In the case of the cell line no differences between samples were noticed, which is caused by indirect contact between samples and cells.

To verify the impact of the scaffolds on the cell morphology, additional studies were carried out (Figure 12). It can be observed that after seven days of primary cell culture, the fibroblasts created a uniform sheet and are interconnected with an extracellular matrix. Their size and shape are typical for normal cells (spindle, longitudinal shape and flat, oval nuclei), and no grains in the cytoplasm are present. The highest cell density is observed in the case of nanomaterials containing nanogold (CS-PLA-30-Au and CS-PLA-35-Au) and pure fibers (CS-PLA-30 and CS-PLA-35). Primary cells are much more vulnerable than cells from the cell line and exhibit the highest resemblance in the biological responses to the in vivo conditions. The HDF cells are present at the bottom of the multi-whole plate and also proliferate under the biomaterials or right next to them, and some interactions may occur between samples and fibroblasts. The smallest cell density can be observed for the CS-PLA-35-ZnO sample, which can be explained by too high ZnO NPs content. Nevertheless, all of the scaffolds do not affect negative cells morphology or proliferating activity. Thus, the cytotoxicity level of the prepared biomaterials can be considered as 0, which means that they can be considered as biosafe.

**Figure 13:**
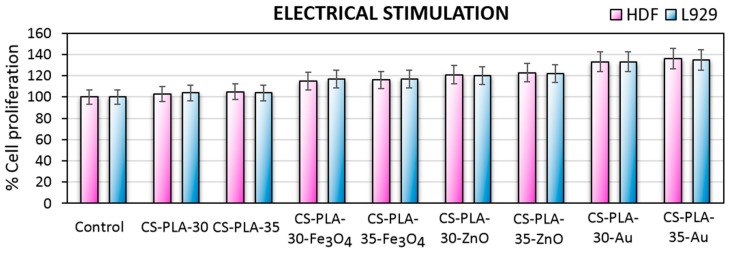
Metabolic activity of L929 mouse fibroblasts cell line and human dermal fibroblasts (primary cells) after electro-stimulated cell culture.

Tissue engineering aims to help damaged organs to regenerate, thus the bioactivity of scaffolds is a highly desired feature. One of the most promising methods for skin wound healing acceleration is electrostimulation. Figure 13 presents results of the electro-stimulated cell culture of the human dermal fibroblast, which are primary cells and mouse L929 fibroblasts cell line. In all cases, direct current application positively affects cells proliferation, which corresponds to other researchers’ results [43,44,45]. Moreover, the efficiency of stimulation depends on the nanoparticles type present in the scaffold, since it was shown in Figure 4 that they display different conductivity. Studies carried out on human dermal fibroblasts by Rouabhia et al. [29] showed that cells viability depends on the stimulation time and have a positive impact on their number (up to 35%; 50 mV/mm). Obtained data presented in Figure 10 corresponds to aforementioned studies since the increase in cells proliferation ranges from 8–36% depending on the cells and scaffold used for the experiment [29].

### 3.8. Fibroblast Specific Protein Study

**Figure 14:**
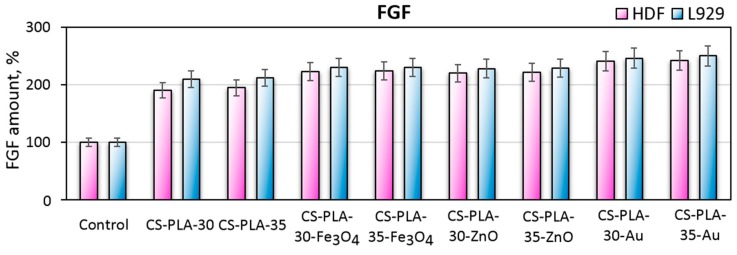
Basic Fibroblast Growth Factor secreted by L929 fibroblasts and human dermal fibroblasts.

Fibroblasts, which are known to be the most abundant cells in both dermis and connective tissue in general, play a crucial role in the skin regeneration process due to their ability of various important biomolecules including growth factors such as insulin like growth factor-1, keratinocyte growth factor, transforming growth factor-beta, platelets derived growth factor and finally basic fibroblast growth factor abbreviated as bFGF [27]. Generally, fibroblast growth factor is secreted by fibroblasts during wound regeneration process. Basic FGF is responsible for tissue growth, angiogenesis, and differentiation and is one of the marker proteins of fibroblasts responsible for cells proliferation [27]. Figure 14 presents the results of bFGF determination during cell culture of human dermal fibroblasts and the L929 mouse fibroblasts cell line. Both primary cells and cell lines secrete bFGF, and the amount of the protein is doubled comparing to the control (2D cell culture). Such results correspond to the metabolic activity presented in Figure 10. It can be observed that mouse fibroblasts exhibit higher FGF secretion levels comparing to primary cells and that higher FGF levels appear for samples with nanofibers higher density and lower diameter (PLA NFs prepared under 35 kV). Also, the highest bioactivity was achieved in the case of samples containing nanogold, namely CS-PLA-30-Au (246%) and CS-PLA-35-Au (250%), which can be assigned to positive impact of Au NPs on cell life cycles [23]. Such results correspond to other researchers’ data, which means that newly developed scaffolds do not change fibroblasts phenotype due to the presence of marker proteins typical for these cells [27].

## 4. Conclusions

In this paper, a novel type of hybrid bioactive scaffold is presented that is based on PLA nanofibers, acylated chitosan, and nanoparticles with conductive properties. The studies performed confirmed nanofibrous and the highly porous architecture of the composites functionalized with ZnO, Fe_3_O_4_, and Au nanoparticles with tailored properties. Further investigations on their water vapor permeability, susceptibility to biodegradation, and conductivity prove that these hybrid materials meet the requirements for skin tissue scaffolds that promote the wound healing process. Finally, their lack of cytotoxicity and bioactivity has been shown on both fibroblasts cell line and primary cells. Moreover, it was proven that the application of electro-stimulation enhances cell proliferation. Taking together, the proposed nanomaterials display very interesting properties and may be considered as very promising biomaterials in terms of regenerative medicine, especially in the case of full thickness skin burns requiring grafts.

## Figures and Tables

**Table 1:** Scaffolds composition.

Sample	Acetylated CS, g	NPs Type, %	PLA, %	Solvent	Potential, kV
CS-PLA-30	0.5	-	10	acetone	30
CS-PLA-35		-			35
CS-PLA-30-Fe_3_O_4_		Fe_3_O_4_, 1.0			30
CS-PLA-35-Fe_3_O_4_		Fe_3_O_4_, 1.0			35
CS-PLA-30-ZnO		ZnO, 1.0			30
CS-PLA-35-ZnO		ZnO, 1.0			35
CS-PLA-30-Au		Au, 1.0			30
CS-PLA-35-Au		Au, 1.0			35

## References

[1] Rowan M.P., Cancio L.C., Elster E.A., Burmeister D.M., Rose L.F., Natesan S., Chan R.K., Christy R.J., Burn K.C. (2015). Wound healing and treatment: Review and advancements. Crit Care.

[2] Chen S., Liu B., Carlson M.A., Gombart A.F., Reilly D.A., Xie J. (2017). Recent advances in electrospun nanofibers for wound healing. Nanomedicine.

[3] Olczyk P., Mencner Ł., Komosinska-Vassev K. (2014). The Role of the Extracellular Matrix Components in Cutaneous Wound Healing. BioMed Res. Int..

[4] Palo M., Rönkönharju S., Tiirik K., Viidik L., Sandler N., Kogermann K. (2019). Bi-Layered Polymer Carriers with Surface Modification by Electrospinning for Potential Wound Care Applications. Pharmaceutics.

[5] Rogina A. (2014). Electrospinning process: Versatile preparation method for biodegradable and natural polymers and biocomposite systems applied in tissue engineering and drug delivery. Appl. Surf. Sci..

[6] Cassan D., Becker A., Glasmacher B., Roger Y., Hoffmann A., Gengenbach T.R., Easton C.D., Hänsch R., Menzel H. (2019). Blending chitosan-g-poly(caprolactone) with poly(caprolactone) by electrospinning to produce functional fiber mats for tissue engineering applications. J. Appl. Polym. Sci..

[7] Yusof M.R., Shamsudin R., Zakaria S., Hamid M.A.A., Yalcinkaya F., Abdullah Y., Yacob N. (2019). Fabrication and Characterization of Carboxymethyl Starch/Poly(l-Lactide) Acid/β-Tricalcium Phosphate Composite Nanofibers via Electrospinning. Polymers.

[8] Chan K.V., Asadian M., Onyshchenko I., Declercq H., Morent R., De Geyter N. (2019). Biocompatibility of Cyclopropylamine-Based Plasma Polymers Deposited at Sub-Atmospheric Pressure on Poly (ε-caprolactone) Nanofiber Meshes. Nanomaterials.

[9] Cavanaugh M., Silantyeva E., Koh G.P., Malekzadeh E., Lanzinger W.D., Willits R.K., Becker M.L. (2019). RGD-Modified Nanofibers Enhance Outcomes in Rats after Sciatic Nerve Injury. J. Funct. Biomater..

[10] Bhattarai R.S., Bachu R.D., Boddu S.H.S., Bhaduri S. (2019). Biomedical Applications of Electrospun Nanofibers: Drug and Nanoparticle Delivery. Pharmaceutics.

[11] Niiyama E., Uto K., Lee C.M., Sakura K., Ebara M. (2018). Alternating Magnetic Field-Triggered Switchable Nanofiber Mesh for Cancer Thermo-Chemotherapy. Polymers.

[12] Ishikawa S., Iijima K., Sasaki K., Hashizume M., Kawabe M., Otsuka H. (2018). Cartilage Differentiation of Bone Marrow-Derived Mesenchymal Stem Cells in Three-Dimensional Silica Nonwoven Fabrics. Appl. Sci..

[13] Szentivanyi A.L., Zernetsch H., Menzel H., Glasmacher B. (2011). A review of developments in electrospinning technology: New opportunities for the design of artificial tissue structures. Int. J. Artif. Organs.

[14] Dash M., Chiellini F., Ottenbrite R.M., Chiellini E. (2011). Chitosan—A versatile semi-synthetic polymer in biomedical applications. Prog. Polym. Sci..

[15] Shahidi F., Abuzaytoun R. (2005). Chitin, chitosan, and co-products: Chemistry, production, applications, and health effects. Adv. Food Nutr. Res..

[16] Kumar P.T., Lakshmanan V.K., Anilkumar T.V., Ramya C., Reshmi P., Unnikrishnan A.G., Nair S.V., Jayakumar R. (2012). Flexible and microporous chitosan hydrogel/nano ZnO composite bandages for wound dressing: In vitro and in vivo evaluation. ACS Appl. Mater. Interfaces.

[17] Kean T., Thanou M. (2010). Biodegradation, biodistribution and toxicity of chitosan. Adv. Drug Deliv. Rev..

[18] Fathi-Achachelouei M., Knopf-Marques H., Ribeiro da Silva C.E., Barthès J., Bat E., Tezcanerand A., Vrana N.E. (2019). Use of Nanoparticles in Tissue Engineering and Regenerative Medicine. Front. Bioeng. Biotechnol..

[19] Wang X., Cheng F., Gao J., Wang L. (2015). Antibacterial wound dressing from chitosan/polyethylene oxide nanofibers mats embedded with silver nanoparticles. J. Biomater. Appl..

[20] AshaRani P.V., Low Kah Mun G., Hande M.P., Valiyaveettil S. (2009). Cytotoxicity and genotoxicity of silver nanoparticles in human cells. ACS Nano.

[21] Chithrani B.D., Ghazani A.A., Chan W.C. (2006). Determining the size and shape dependence of gold nanoparticle uptake into mammalian cells. Nano Lett..

[22] Chompoosor A., Saha K., Ghosh P.S., Macarthy D.J., Miranda O.R., Zhu Z.J., Arcaro K.F., Rotello V.M. (2010). The role of surface functionality on acute cytotoxicity, ROS generation and DNA damage by cationic gold nanoparticles. Small.

[23] Zhang M., Chen S., Zhong L., Wang B., Wang H., Hong F. (2019). Zn2+-loaded TOBC nanofiber-reinforced biomimetic calcium alginate hydrogel for antibacterial wound dressing. Int. J. Biol. Macromol..

[24] Jatoi A.W., Kim I.S., Ogasawarad H., Ni Q.Q. (2019). Characterizations and application of CA/ZnO/AgNP composite nanofibers for sustained antibacterial properties. Mater. Sci. Eng. C.

[25] Wang W., Zheng T., Sheng B., Zhou T., Zhang Q., Wu F., Zhou N., Shen J., Zhang M., Sun Y. (2019). Functionalization of polyvinyl alcohol composite film wrapped in am-ZnO@CuO@Au nanoparticles for antibacterial application and wound healing. Appl. Mater. Today.

[26] Cai N., Li C., Han C., Luo X., Shen L., Xue Y., Yu F. (2016). Tailoring mechanical and antibacterial properties of chitosan/gelatin nanofiber membranes with Fe_3_O_4_ nanoparticles for potential wound dressing application. Appl. Surf. Sci..

[27] Behera S.S., Das U., Kumar A., Bissoyi A., Singh A.K. (2017). Chitosan/TiO_2_ composite membrane improves proliferation and survival of L929 fibroblast cells: Application in wound dressing and skin regeneration. Int. J. Biol. Macromol..

[28] Radwan-Pragłowska J., Piątkowski M., Janus Ł., Bogdał D., Matysek D., Cablik V. (2019). 3D scaffolds prepared from acylated chitosan applicable in skin regeneration—Synthesis and characterization. Int. J. Polym. Anal. Charact..

[29] Rouabhia M., Park H., Meng S., Derbali H., Zhang Z. (2013). Electrical Stimulation Promotes Wound Healing by Enhancing Dermal Fibroblast Activity and Promoting Myofibroblast Transdifferentiation. PLoS ONE.

[30] Sluzky V., Shahrokh Z., Stratton P., Eberlein G., Wang Y.J. (1994). Chromatographic Methods for Quantitative Analysis of Native, Denatured, and Aggregated Basic Fibroblast Growth Factor in Solution Formulations. Pharm. Res..

[31] Coltelli M.B., Cinelli P., Gigante V., Aliotta L., Morganti P., Panariello L., Lazzeri A. (2019). Chitin Nanofibrils in Poly(Lactic Acid) (PLA) Nanocomposites: Dispersion and Thermo-Mechanical Properties. Int. J. Mol. Sci..

[32] Ambekar R.S., Kandasubramanian B. (2019). Advancements in nanofibers for wound dressing: A review. Eur Polym. J..

[33] Santoro M., Shah S.R., Walker J.L., Mikos A.G. (2016). Poly(lactic acid) nanofibrous scaffolds for tissue engineering. Adv. Drug Deliv. Rev..

[34] Zhao X., Gao J., Hu X., Guo H., Wang F., Qia Y., Wang L. (2018). Collagen/Polyethylene Oxide Nanofibrous Membranes with Improved Hemostasis and Cytocompatibility for Wound Dressing. J. Appl. Sci..

[35] Massoumi B., Abbasian M., Jahanban-Esfahlan R., Mohammad-Rezaei R., Khalilzadeh B., Samadian H., Rezaei A., Derakhshankhah H., Jaymand M. (2019). A novel bio-inspired conductive, biocompatible, and adhesive terpolymer based on polyaniline, polydopamine, and polylactide as scaffolding biomaterial for tissue engineering application. Int. J. Biol. Macromol..

[36] Zarrintaj P., Rezaeian I., Bakhshandeh B., Heshmatian B., Ganjali M.R. (2017). Bio-Conductive Scaffold Based on Agarose-Polyaniline for Tissue Engineering. J. Skin Stem Cell.

[37] Talikowska M., Fu X., Lisak G. (2019). Application of conducting polymers to wound care and skin tissue engineering: A review. Biosens. Bioelectron..

[38] Sun Y.S. (2017). Electrical Stimulation for Wound-Healing: Simulation on the Effect of Electrode Configurations. BioMed Res. Int..

[39] Tang P., Han L., Li P., Jia Z., Wang K., Zhang H., Tan H., Guo T., Lu X. (2019). Mussel-Inspired Electroactive and Antioxidative Scaffolds with Incorporation of Polydopamine-Reduced Graphene Oxide for Enhancing Skin Wound Healing. ACS Appl. Mater. Interfaces.

[40] Xu R., Xia H., He W., Li Z., Zhao J., Liu B., Wang Y., Lei Q., Kong Y., Bai Y. (2016). Controlled water vapor transmission rate promotes wound-healing via wound re-epithelialization and contraction enhancement. Sci. Rep..

[41] Lima L.L., Taketa T.B., Beppu M.M., De Oliveira Sousa I.M., Foglio M.A., Moraes A.M. (2019). Coated electrospun bioactive wound dressings: Mechanical properties and ability to control lesion microenvironment. Mater. Sci. Eng. C.

[42] Zhao G., Bao X., Huang G., Xu F., Zhang X. (2019). Differential Effects of Directional Cyclic Stretching on the Functionalities of Engineered Cardiac Tissues. ACS Appl. Bio Mater..

[43] Qu J., Zhao X., Liang Y., Xu Y., Ma P.X., Guo B. (2019). Degradable conductive injectable hydrogels as novel antibacterial, anti-oxidant wound dressings for wound healing. Chem. Eng. J..

[44] Zhao G., Qing H., Huang G., Genin G.M., Lu T.J., Luo Z., Xu F., Zhang X. (2018). Reduced graphene oxide functionalized nanofibrous silk fibroin matrices for engineering excitable tissues. NPG Asia Mater..

[45] Zhao G., Zhang X., Lu T.J., Xu F. (2015). Recent Advances in Electrospun Nanofibrous Scaffolds for Cardiac Tissue Engineering. Adv. Funct. Mater..

